# Molecular analysis of *DHFR* and *DHPS* gene mutations in *Plasmodium cynomolgi* from humans and macaques in Southeast Asia

**DOI:** 10.1051/parasite/2025057

**Published:** 2025-09-29

**Authors:** Raweewan Sangsri, Nathjanan Jongkon, Kiattawee Choowongkomon, Suchinda Malaivijitnond, Nicholas P.J. Day, Arjen M. Dondorp, Mallika Imwong

**Affiliations:** 1 Department of Molecular Tropical Medicine and Genetics, Faculty of Tropical Medicine, Mahidol University Bangkok 10400 Thailand; 2 Department of Social and Applied Science, College of Industrial Technology, King Mongkut’s University of Technology North Bangkok Bangkok 10800 Thailand; 3 Department of Biochemistry, Faculty of Science, Kasetsart University Bangkok 10903 Thailand; 4 National Primate Research Center of Thailand, Chulalongkorn University Saraburi 18110 Thailand; 5 Department of Biology, Faculty of Science, Chulalongkorn University Bangkok 10330 Thailand; 6 Mahidol-Oxford Tropical Medicine Research Unit, Faculty of Tropical Medicine, Mahidol University Bangkok 10400 Thailand; 7 Centre for Tropical Medicine and Global Health, Nuffield Department of Medicine, University of Oxford Old Road Campus Oxford OX3 7LF UK

**Keywords:** *Plasmodium cynomolgi*, *Pcydhfr*, *Pcydhps*, Simian malaria

## Abstract

*Plasmodium cynomolgi* is an emerging zoonotic malaria parasite in Southeast Asia, infecting both humans and macaques. In this study, we investigated mutations in the *DHFR* and *DHPS* genes of *P. cynomolgi* from humans and macaques, comparing them to known resistance mutations in *P. falciparum* and *P. vivax*. We also examined how these mutations affect antifolate drug binding, which may influence treatment efficacy and resistance. Nine asymptomatic human blood samples from Cambodia and 29 macaque samples from Thailand were analyzed. Human samples included eight *P. cynomolgi* monoinfections and one mixed infection with *P. vivax*, while all macaque samples were monoinfections. The *PcyDHFR* and *PcyDHPS* genes were amplified, sequenced, and subjected to haplotype analysis. Human samples from Battambang, Cambodia were 100% identical to the *P. cynomolgi* RO strain, showing no *DHFR* mutations and one *DHPS* mutation (V451I). In contrast, macaque samples from Saraburi, Thailand showed *PcyDHFR* mutations N44T and C49S, and two haplotypes based on I7 variation – haplotype 1 (72.41%) with wild-type I7 and haplotype 2 (27.59%) with the I7 mutation. *PcyDHPS* mutations were identical across macaque isolates. Protein structures of *Pcy*DHFR and *Pcy*DHPS were modeled using SWISS-MODEL, focusing on the N- and C-terminals. Mutations occurred near catalytic sites but did not significantly affect binding affinity, based on molecular docking with eight antifolate drugs. These findings suggest that current antifolate drugs remain potentially effective against *P. cynomolgi*, and highlight the importance of monitoring drug resistance in zoonotic malaria.

## Abstract (fr)

*Plasmodium cynomolgi* est un parasite zoonotique du paludisme émergent en Asie du Sud-Est, infectant à la fois l’homme et le macaque. Cette étude a examiné les mutations des gènes *DHFR* et *DHPS* de *P. cynomolgi* chez l’homme et le macaque, en les comparant aux mutations de résistance connues chez *P. falciparum* et *P. vivax*. Nous avons également examiné l’impact de ces mutations sur la liaison aux antifolates, ce qui pourrait influencer l’efficacité et la résistance au traitement. Neuf échantillons de sang humain asymptomatique provenant du Cambodge et 29 échantillons de sang de macaques provenant de Thaïlande ont été analysés. Les échantillons humains comprenaient huit mono-infections à *P. cynomolgi* et une infection mixte à *P. vivax*, tandis que tous les échantillons de macaques étaient des mono-infections. Les gènes *PcyDHFR* et *PcyDHPS* ont été amplifiés, séquencés et soumis à une analyse d’haplotype. Les échantillons humains de Battambang, au Cambodge, étaient identiques à 100 % à la souche RO de *P. cynomolgi*, ne présentant aucune mutation *dhfr* et une mutation *dhps* (V451I). En revanche, les échantillons issus de macaques de Saraburi, en Thaïlande, présentaient les mutations N44T et C49S du gène *PcyDHFR*, ainsi que deux haplotypes basés sur la variation I7 : l’haplotype 1 (72,41 %) avec le gène I7 sauvage et l’haplotype 2 (27,59 %) avec la mutation I7. Les mutations du gène *PcyDHPS* étaient identiques entre les isolats de macaques. Les structures protéiques de *Pcy*DHFR et de *Pcy*DHPS ont été modélisées à l’aide de SWISS-MODEL, en se concentrant sur les extrémités N et C. Les mutations se sont produites à proximité des sites catalytiques, mais n’ont pas eu d’effet significatif sur l’affinité de liaison, d’après l’amarrage moléculaire avec huit médicaments antifolates. Ces résultats suggèrent que les médicaments antifolates actuels restent potentiellement efficaces contre *P. cynomolgi* et soulignent l’importance de surveiller la résistance aux médicaments dans le paludisme zoonotique.

## Introduction

Malaria is a major global health concern, particularly in tropical and subtropical regions, where over 200 million cases are reported annually. While *Plasmodium falciparum* and *Plasmodium vivax* are the most well-known species affecting humans, recent attention has turned toward zoonotic malaria, specifically infections caused by *P. cynomolgi*, a parasite that primarily infects macaques. Evidence has emerged of its ability to infect humans, although infections are often asymptomatic or mild. Nevertheless, it is a potential emerging zoonotic threat, especially in Southeast Asia, where human populations areas frequently overlap with primate habitats [16, 19]. In Cambodia and Thailand, both human and macaque populations carry *P*. *cynomolgi*; thus, these regions are key areas for studying the zoonotic transmission dynamics of malaria [37]. Given the increasing proximity between humans and wildlife due to habitat encroachment, closely monitoring these zoonotic pathogens is critical. Furthermore, as drug-resistant strains of malaria parasites become more prevalent, understanding the genetic mutations in *P*. *cynomolgi* that contribute to drug resistance is essential for developing future therapeutic strategies [26].

The dihydrofolate reductase (*DHFR*) and dihydropteroate synthase (*DHPS*) genes play pivotal roles in the development of antifolate drug resistance in malaria parasites, including *P*. *cynomolgi*. The *DHFR* gene encodes an enzyme that reduces dihydrofolate to tetrahydrofolate, which is essential for DNA synthesis. Mutations in *DHFR*, such as N51I, C59R, S108N, and I164L, reduce the effectiveness of antifolate drugs such as pyrimethamine by altering the enzyme’s binding site in a manner that enables drug resistance [7]. Similarly, in the *DHPS* gene (which codes for an enzyme involved in folate biosynthesis), mutations at key positions, such as A437G and K540E, disrupt drug binding and confer resistance to sulfa drugs such as sulfadoxine and sulfamethoxazole [33]. Drug resistance due to these mutations is particularly concerning, because it can lead to treatment failure with sulfadoxine-pyrimethamine (SP) combination therapy, as has been seen in human malaria parasites [26]. Moreover, these mutations in *P*. *cynomolgi* raise concerns about zoonotic malaria transmission and the spread of drug-resistant strains from macaques to humans.

The main research questions for this study focus on understanding the genetic and molecular mechanisms of *P*. *cynomolgi* drug resistance. To accomplish this, we first identified the genetic mutations in the *DHFR* and *DHPS* genes in both human and macaque populations and compared these mutations to those found in *P*. *falciparum* and *P*. *vivax*. Second, we investigated how these mutations affect the binding affinity of antifolate drugs to the *Pcy*DHFR and *Pcy*DHPS proteins, thus determining whether these mutations contribute to drug resistance. Lastly, we examined the prevalence of these mutations in different host populations to assess how genetic variability may influence zoonotic transmission and the effectiveness of treatment strategies for *P*. *cynomolgi* infections. By combining field sample collection with cutting-edge molecular techniques, the results of this study provide a deeper understanding of the genetic diversity and drug resistance mechanisms in *P*. *cynomolgi*, which will aid in the development of more effective treatment and prevention strategies for zoonotic malaria.

## Materials and methods

### Ethics approval

This study involving humans was approved by the ethics review committees of the Faculty of Tropical Medicine, Mahidol University (approval number: MUTM2023-015-02), while a protocol for macaque blood collecting, processing, and handling was approved by the Animal Care and Use Committees of the National Primate Research Center of Thailand-Chulalongkorn University (Protocol Review No. 2075007) and Institutional Animal Care and Use Committee, Faculty of Tropical Medicine, Mahidol University (FTM-ACUC 013/2022E) to ensure the well-being of macaques during the study.

### Studied sites and sample collections

The nine human venous blood samples of asymptomatic malaria were collected between 2015 and 2016 from the Battambang province of Cambodia. Eight samples exhibited *P*. *cynomolgi* monoinfection, while 1 sample showed mixed infection with *P*. *vivax* [8]. Additionally, 29 EDTA-blood samples were collected from long-tailed macaques (*Macaca fascicularis*) in 2015 at Wat Tham Phrapothisat, Saraburi Province, Thailand. The infected macaques were identified to have mono *P*. *cynomolgi* infection [13]. All 200 μL of blood samples were extracted using a QIAamp^®^ DNA Mini kit (QIAGEN, Hilden, Germany), following the manufacturer’s instructions. The extracted DNA was stored at −20 °C for further study. The simian *Plasmodium* species were identified using a PCR protocol targeting *18S rRNA* and *cox1* gene [8, 24] which were designed to cover a conserved genes region among those species.

### *Pcydhps* amplification and *pcydhfr*-*pcydhps* haplotype analysis

The reference sequence used throughout the manuscript refers to the *Plasmodium cynomolgi* M strain (Gene number on PlasmoDB database: PcyM_0526900 for *pcydhfr*; PcyM_1430900 for *pcydhps*), which serves as the standard for primer design, sequence alignment and variant calling. The five interesting *Pcy*DHPS positions that aligned to be equal to the *Pf*/*Pv*DHPS binding pocket were amplified by newly designed primers based on the reference sequence of *P*. *cynomolgi* strain M by Primer3Plus (https://www.primer3plus.com/index.html). There was no cross-reaction with other species except *P*. *inui*, a striking similarity of approximately 91% in nucleotide and amino acid sequences. *Pcydhps* was amplified and confirmed PCR product by sequencing. The primers and *pcydhps* gene amplifying profile are shown in Table 1. The nucleotide and amino acid sequences of *P*. *cynomolgi* in this study were confirmed by running an NCBI BLAST search. The *pcydhps* mutation was evaluated by alignment against the reference sequence, *P*. *cynomolgi* strain M (gene locus: PCYM_1430900) using MEGA11 software [31]. The wild-type sequence, for the purpose of our analysis, refers to the most prevalent allele observed among *P*. *cynomolgi* isolates from macaques in previous studies and is consistent with the non-mutated (non-resistant) phenotype at the key loci examined. A haplotype pattern of *pcydhfr*-*pcydhps* was analyzed by combining a previous *pcydhfr* of human [8] and macaque *P*. *cynomolgi* [13] with the result of *pcydhps* in this study.

**Table 1:** Primers and PCR conditions to amplify *pcydhps*.

Primer name	Binding location in Chromosome 14	Sequence (5’→3’)	PCR conditions	Product (bp)
PcyDHPS_N1F:	13303-1…1330273 (–)	GGCTATACGTATTGAAAGATAAAGTATCA	94°C for 30 s; 50°C for 30 s;	838
PcyDHPS_R:	1329480…1329464 (–)	TCGAAGCCCCCATTGGT	72°C for 1 min for 35 cycles.	
PcyDHPS_N2F:	1330185…1330164 (–)	AAGCTGTGGAAAGGATGTTCG	94°C for 30 s; 57°C for 30 s;	721
			72°C for 1 min for 30 cycles.	

### Homology protein modelling and molecular docking

Only the *Pcy*DHFR (1st–244th) and *Pcy*DHPS (334th–724th) domains of the proteins were used to build 3D protein structures, based on sequence similarity on the SWISS-MODEL (https://swissmodel.expasy.org/interactive) with the default setting. The protein sequences in FASTA format were inputted into the browser, and the resulting 3D protein structures were retrieved in PDB format for further analysis with molecular docking and molecular dynamics.

The built protein structures were analyzed by molecular docking using the Genetic Optimisation for Ligand Docking (GOLD) program [12], a widely recognized tool in the field. Six inhibitors were used for *Pcy*DHFR analysis: pyrimethamine (PYR), cycloguanil (1CY), trimethoprim (TOP), P218, P65, and WR99210. Meanwhile, the *Pcy*DHPS structures were performed with the natural substrate, 4-aminobenzoic acid (*p*ABA), and its inhibitors are sulfadoxine (SDX) and sulfamethoxazole (SMZ). The 3D structure of these ligands was loaded from PubChem. The ligands of *Pcy*DHFR and *Pcy*DHPS were docked within 6 and 3 Å of the protein binding pocket, respectively, and run with a genetic algorithm 100 times without early termination. The best poses of protein docking were selected based on the highest ASP scoring function for both *Pcy*DHFR and *Pcy*DHPS; the ASP scoring of *Pcy*DHPS was re-scored from Chem to ASP score to ensure greater accuracy. BIOVIA Discovery Studio Visualizer V21.1.0 [1], a powerful software for interaction analysis, was used to analyze the best poses protein complex. All 3D structures and interactions were visualized using a PYMOL Molecular Graphics System, V2.5.2 [28].

### Molecular dynamics (MD) simulation and binding free energy calculations

We used MD simulation to evaluate the stability of a protein-ligand system. Protein-ligand complexes for the best-ranked molecules obtained by GOLD docking were each subjected to a 100-ns MD simulation using GROMACS with the Gromos54a7 forcefield (FF). All ligand topology files were obtained from the Automated Topology Builder (ATB) server using the “all atoms” option [17]. The initial coordinates of the GOLD-docked structures were used as the initial structures for the MD simulations. The structures were solvated in a cubic box of SCP waters with a 1 nm distance from the protein to the edge of the box. The 0.15 M concentration of Na^+^ and Cl^−^ ions was added to neutralize the complex systems. The minimization steps were subjected to 50,000 to reduce incorrect interatomic contacts by the steepest-descent minimization method before proceeding to the other two equilibration steps. The first step was done in the NVT (5 ns) ensemble by gradually heating the systems to 300 K with a time-step of 2 femtoseconds, and the second step was done at a pressure of 1 atm in the NPT (5 ns) ensemble. 100 ns of triplicates running MD with a time-step of 2 fs were done on each system. The stability and fluctuation of the *Pcy*DHFR-PYR complexes were monitored using the averaged RMSD calculation of the protein backbone and ligand plotted along a simulation time of 100 ns. The 81–100 ns of simulation time were selected to determine hydrogen bond and percent occupancy using 3.5 Å between donor-acceptor and angle cutoff of the 30-degree parameter. A g_mmpbsa tool from GROMACS implemented the Molecular Mechanics Poisson-Boltzmann Surface Area (MM-PBSA) approach to calculate the total binding free energies of the *Pcy*DHFR-PYR complexes in the solvent [6, 30]. The data were analyzed and visualized using RStudio version 4.3.2.

### Statistical analysis

The experiments on binding free energy for all *P*. *cynomolgi* variants were conducted in three replicates. The results were exhibited as mean ± standard deviation (SD), and the Shapiro-Wilk Test was tested to check the normality of the data with RStudio version 4.3.2 [25]. Obtaining a *p*-value of more than 0.05 (*p* > 0.05) implies the data had a normal distribution. All data were further assessed by one-way ANOVA to compare the means of each variant; a *p*-value less than 0.05 (*p* < 0.05) was considered significantly different in mean among variants.

## Results

### Analysis of *pcydhps* sequence and haplotype *pcydhfr*-*pcydhps* of *P*. *cynomolgi* from Cambodia and Thailand

A partial, 721-bp region of *pcydhps* was successfully amplified, sequenced, and aligned against the reference sequence of *P*. *cynomolgi* strain M (gene locus: PcyM_1430900), which contains the complete Open Reading Frame (ORF) of the *DHPS* gene. A missense mutation was detected in 100% (9/9) of human *P*. *cynomolgi* from Battambang, Cambodia, with the mutation at V451I. In contrast, wild Thai long-tailed macaques from Saraburi province were identified with nine mutations at K411N, S414R, A424D, G433A, L444V, V451I, A483E, T497S, and A585V in all macaques isolated samples (100%; 29/29). The mutation at V451I was observed in both isolates. Remarkably, the A585V mutation in wild macaques was equivalent to the position of the well-known drug resistance mutations at A613 and V585 of *P*. *falciparum* and *P*. *vivax*, respectively.

The haplotype *pcydhfr*-*pcydhps* was analyzed by combining the *pcydhps* polymorphism in this study with a previous publication of *pcydhfr* in humans from Cambodia [8] and wild macaques [13] from Thailand. Table 2 presents a comparative analysis of amino acid mutations in the DHFR and DHPS domains across *P*. *falciparum*, *P*. *vivax*, and *P*. *cynomolgi* strains (M, B, RO) alongside the haplotype pattern identified in this study. The human isolates exhibited a single haplotype pattern of *pcydhfr*-*pcydhps*, characterized by quadruple mutations of *pcydhfr* at C49G, F79Y, G162D, and S205A, and a single mutation at V451I of *pcydhps*.

**Table 2:** Comparison analysis of polymorphic alleles *Pcy*DHFR and *Pcy*DHPS. The bold blue text indicates different residues among three strains of *P*. *cynomolgi*. Bold text represents the polymorphic alleles *Pcy*DHFR and *Pcy*DHPS in this study.

	Equivalent residues in human malaria		*P. cynomolgi* strains		*P*. *cynomolgi* in this study			
			
			Available on database	Reference	Residue numbering based on the reference sequence strain M	*Homo sapiens* (*n* = 9)	*Macaca fascicularis* (*n* = 29)
			
	*P*. *falciparum* strain 3D7	*P*. *vivax* strain SalI	Strain RO^1^	Strain B^2^/strain M^3^		Battambang, Cambodia	Saraburi, Thailand	Saraburi, Thailand
						Haplotype 1 100% (9/9)	Haplotype 1 72.41% (21/29)	Haplotype 2 27.59% (8/29)
Active site of *Pcy*DHFR [8, 13]	A16	A15	A15	A	A15	•	•	•
	N51	N50	N50	N	N50	•	•	•
	F58	F57	F57	F	F57	•	•	•
	C59	S58	S58	S	S58	•	•	•
	S108	S117	S123	S	S123	•	•	•
	I164	I173	I179	I	I179	•	•	•
*Pcy*DHFR nonsynonymous mutations [8, 13]	V8	V7	–	I	I7	•	•	V*
	K23	T22	S22	S	S22	•	**P**	**P**
	N24	S23	N23	N	N23	•	**S**	**S**
	N34	P33	S33	S	S33	•	**P**	**P**
	V45	T44	N44	N	N44	•	**T**	**T**
	C50	C49	**G49**	**C**	C49	**G**	**S**	**S**
	Y80	Y79	**Y79**	**F**	F79	**Y**	**Y**	**Y**
	K83	M82	R82	R	R82	•	**K**	**K**
	–	D90	D90	D	D90	•	**G**	**G**
	–	T92	H92	H	H92	•	**L**	**L**
	–	G94	G94	G	G94	•	**S**	**S**
	–	–	T97	T	T97	•	**I**	**I**
	–	–	N98	N	N98	•	**S**	**S**
	N88	N97	A103	A	A103	•	**T**	**T**
	D91	G100	T106	T	T106	•	**I**	**I**
	N94	N103	N109	N	N109	•	**S**	**S**
	S95	A104	N110	N	N110	•	**T**	**T**
	K133	K142	Y148	Y	Y148	•	**H**	**H**
	D135	D144	H150	H	H150	•	**Q**	**Q**
	Y141	F150	F156	F	F156	•	**Y**	**Y**
	E147	D156	**D162**	**G**	G162	**D**	**D**	**D**
	N157	K166	R172	R	R172	•	**K**	**K**
	Q171	R180	R186	R	R186	•	**G**	**G**
	T190	A199	**A205**	**S**	S205	**A**	**A**	**A**
Active site of *Pcy*DHPS	S436	S382	NA	S382	S382	•	•	•
	A437	A383	NA	A383	A383	•	•	•
	K540	K512	NA	K512	K512	•	•	•
	A581	A553	NA	A553	A553	•	•	•
	A613	V585	NA	A585	A585	•	**V**	**V**
*Pcy*DHPS nonsynonymous mutations	D465	K411	NA	K411	K411	•	**N**	**N**
	N468	C414	NA	S414	S414	•	**R**	**R**
	–	A424	NA	A424	A424	•	**D**	**D**
	–	A433	NA	G433	G433	•	**A**	**A**
	–	V444	NA	L444	L444	•	**V**	**V**
	I479	I451	NA	V451	V451	**I**	**I**	**I**
	P511	P483	NA	A483	A483	•	**E**	**E**
	S525	S497	NA	T497	T497	•	**S**	**S**

Note: The strain M was referred to as a wild-type sequence in this context because this strain provides a complete sequence of the *pcydhfr* and *pcydhps* on the PlasmoDB database. ^1^Strain RO from *Macaca mulatta* isolated in 1960 has *DHFR* accession number AY639976.1, while the *DHPS* sequence is unavailable. ^2^Strain B from *Macaca fascicularis* isolated in 1959 has accession number NC_020398.1 for *DHFR*, and NC_020407.1 for *DHPS*. ^3^Strain M from *Macaca fascicularis* isolated in 1933 has gene number PcyM_0526900 for *DHFR*, and NC_020407.1 for *DHPS* on the PlasmoDB database. • Indicates identical amino acid residues to the wild-type sequence. *Containing a mixed genotype changed nucleotide from ATA to A/GTA (*n* = 2 samples).

In contrast, wild macaques were classified into two haplotypes: Haplotype 1, with 72.41%, comprised 23 *pcydhfr* mutations and nine *pcydhps* mutations, while 27.59% of Haplotype 2 contained 24 and 9 mutations of *pcydhfr* and *pcydhps*, respectively (Table 2). These categorized patterns were distinguished by mutation at I7 in *pcydhfr*, with no mutation in Haplotype 1, whereas Haplotype 2 was mutated as altering amino acid from isoleucine to valine (I7V). In addition, the comparison exhibited differences in the sequence of *pcydhfr* in the *P*. *cynomolgi* strain RO that differ from those in other strains at positions C49, F79, G162, and S205, which could suggest a genetic variation in this gene between the strains of *P*. *cynomolgi* in the macaque.

The *pcydhfr* exhibited a key mutation at S33 and C49, which were equivalent to the positions associated with pyrimethamine resistance in *P*. *vivax* and *P*. *falciparum*, respectively. Also, the mutation at A585 of the *pcydhps* was equivalent to one of five key residues in sulfa-drugs resistance in *P*. *vi*vax and *P*. *falciparum*, indicating that these mutations could impact the effectiveness of antifolate drugs in *P*. *cynomolgi* treatment. Therefore, the mutations found in these genes require further analysis by computational protein analysis.

### Homology modeling of *Pcy*DHFR and *Pcy*DHPS proteins

**Figure 1:**
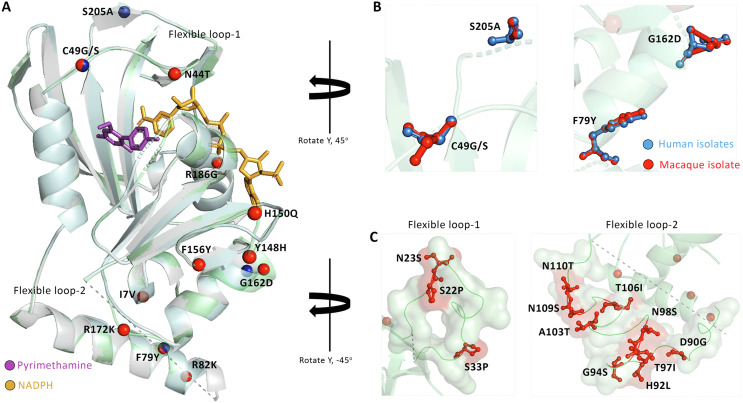
(A) The homologous *Pcy*DHFR protein structure of wild-type (light grey), human (pale cyan), and macaque (pale green) were aligned and shown in a ribbon structure. The mutations in protein were highlighted in the navy and red spheres for human and macaque samples, respectively. (B) Superimposition of four shared mutations between human and macaque *P*. *cynomolgi*. (C) The mutations in disordered protein residues from macaque isolates are shown as red ball and stick. Dashed lines represented a two-flexible loop structure, which is not well defined in the *Pv*DHFR template crystal structure [15], and seven amino acid insertions in a sequence of *Pcy*DHFR protein.

The protein structures of *Pcy*DHFR and *Pcy*DHPS were modeled based on the homology between protein structures using the SWISS-MODEL (https://swissmodel.expasy.org/interactive). To create the 3D structures, a 244-amino acid N-terminal domain of *Pcy*DHFR, equivalent to the 1st–288th and 1st–237th DHFR domains of *P*. *falciparum* and *P*. *vivax*, respectively, was selected. The 3D structures of *Pcy*DHFR were based on the highly relevant *P*. *vivax* DHFR crystal structure (PDB: 2BL9.1) and features a high resolution of 1.90 Å, with a coverage of 98% of the *Pcy*DHFR sequences [15]. The protein sequences of the wild-type, human, and macaque *Pcy*DHFR structures were 83.12%, 84.87%, and 86.13% similar to *Pv*DHFR, respectively. All the build structures contain two flexible loop structures at position 19th–35th and 86th–112th, in which the 12 mutations of macaque isolates comprised three mutations: S22P, N23S, and S33P, which were revealed the position onto Loop-1, and other nine mutations were located on Loop-2 (Fig. 1C). Loop-2 was a repeat region equal to the disordered structure of template *Pv*DHFR, which carried a GGDN tandem repeat region. In the structure of human and macaque variants, the mutated residues near the protein’s catalytic site were detected: C49G for the human variant and C49S and N44T for the macaque variant (Fig. 1A). Thus, the modeled structure of human and macaque variants led to analysis of the effect of these mutations through conduction of molecular docking and interaction with six antimalarial drugs to determine the impact of the mutations on protein-ligand affinity.

**Figure 2:**
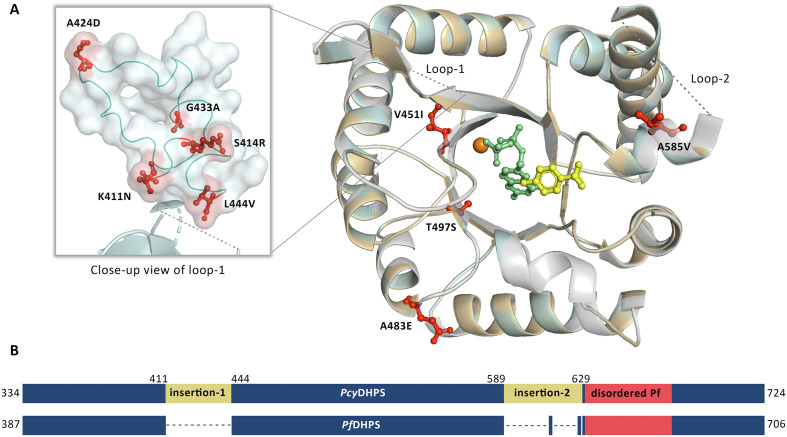
(A) An overlay of the *Pcy*DHPS models of 3 variants: wild-type (light grey), human (light orange), and macaque (pale cyan) isolates. The identified mutations in the study are depicted in a red ball and stick; a small box was zoomed in to view mutations in the region of loop-1. The dashed line indicates loop structures, two long insertion residues in *P*. *cynomolgi*, and a disordered *Pf*DHPS template. The bound substrates *p*ABA (yellow) and PtPP (green) are displayed in a ball and stick representation, while the cofactor magnesium is represented by an orange sphere. (B) Schematic protein sequence alignment of *Pcy*DHPS against *Pf*DHPS, which identified the two insertion sites in the *Pcy*DHPS protein sequence (yellow) and disordered *Pf*DHPS template structure (soft red) [2]. A dash indicates a gap in sequence alignment.

The entire *Pcy*DHPS domain in the range of C-terminal of bifunctional protein, HPPK-DHPS at position 334th–724th was selected for 3D structural modeling based on homology with a *Pf*DHPS template (PDB: 6JWX), which covered 90% of *Pcy*DHPS sequences and shown sequence similarity with structure template were 66.51–66.82% for human and macaque *P*. *cynomolgi*, respectively [2]. The modeled *Pcy*DHPS structures contained two loop structures at 411th–444th and 589th–676th, corresponding to 2 insertion sites in the *Pcy*DHPS sequence compared to the *Pf*DHPS sequence. The Loop-2 were combined from the insertion sequence at 589th–629th, and a not well-defined template *Pf*DHPS structure at position 631st–676th (Fig. 2B). The human *P*. *cynomolgi* isolates carried the V451I mutation that far from the DHPS protein’s active site. In contrast, the macaque isolates presented the A585V mutation, which is equivalent to the protein binding pockets of *Pf*DHPS and *Pv*DHPS at residue A613 and V585, respectively, that are attributed to sulfa-drug resistance in the parasites. Hence, an in-depth analysis of the impact of the detected mutation in this region on protein affinity was conducted on the structure of the macaque isolate by protein docking and interaction analysis using the enzyme’s natural substrate (*p*ABA) and two of its inhibitors, SDX and SMZ.

### Molecular docking between *Pcy*DHFR and its inhibitors

We identified 24 mutations of *Pcy*DHFR in each macaque isolate (Haplotype 2) and 4 mutations from the human isolate, which both variants carrying the C49 mutation, which is located close to the catalytic site of the protein (Fig. 1) and comparable with the C50 mutation associated with PYR resistance in *P*. *falciparum* [3, 5, 11]. However, other identified mutations were included in the structure to determine their potential consequences on protein structure and function by conducting molecular docking with their inhibitors.

The *Pcy*DHFR was docked with three common antimalarial inhibitors, namely PYR, 1CY, and TOP, and we also docked three new antimalarials used for the treatment of resistance *P*. *falciparum*: P218, P65, and WR99210. These inhibitors were determined to bind within 6 Å of the protein’s active site, and reported in the ASP score function and normalized with its molecular weight. The resulting docking and normalized scores show a similarity between mutant and wild-type scores (Table 3). This finding suggests that these drugs were likely to retain the *P*. *cynomolgi* infection therapeutic potential.

**Table 3:** ASP scoring function of molecular docking between *Pcy*DHFR and *Pcy*DHPS and its inhibitors.

Proteins	Substrate/Inhibitors	Variants of *P*. *cynomolgi*			Relative of docking score^1^	
		Wild-type	Human	Macaque	ΔHuman	ΔMacaque
*Pcy*DHFR	Pyrimethamine	37.14 (0.15)	37.46 (0.15)	37.20 (0.15)	−0.32	−0.06
	Cycloguanil	36.52 (0.15)	37.05 (0.15)	36.75 (0.15)	−0.53	−0.23
	Trimethoprim	36.00 (0.12)	36.30 (0.13)	35.45 (0.12)	−0.30	0.55
	P218	49.53 (0.14)	50.33 (0.14)	49.50 (0.14)	−0.80	0.03
	P65	45.87 (0.12)	45.50 (0.12)	45.76 (0.12)	0.37	0.11
	WR99210	44.63 (0.11)	44.21 (0.11)	44.87 (0.11)	0.42	−0.24
*Pcy*DHPS	*p*ABA	13.28 (0.10)	14.02 (0.10)	14.41 (0.11)	−0.74	−1.12
	Sulfadoxine	19.89 (0.06)	22.09 (0.07)	20.94 (0.07)	−2.20	−1.05
	Sulfamethoxazole	16.67 (0.07)	17.63 (0.07)	17.12 (0.07)	−0.96	−0.46

Note: The value in parentheses describes a normalization scoring function by dividing it by the molecular weight of its inhibitors. ^1^represents a relative of its docking score with the wild-type.

**Figure 3:**
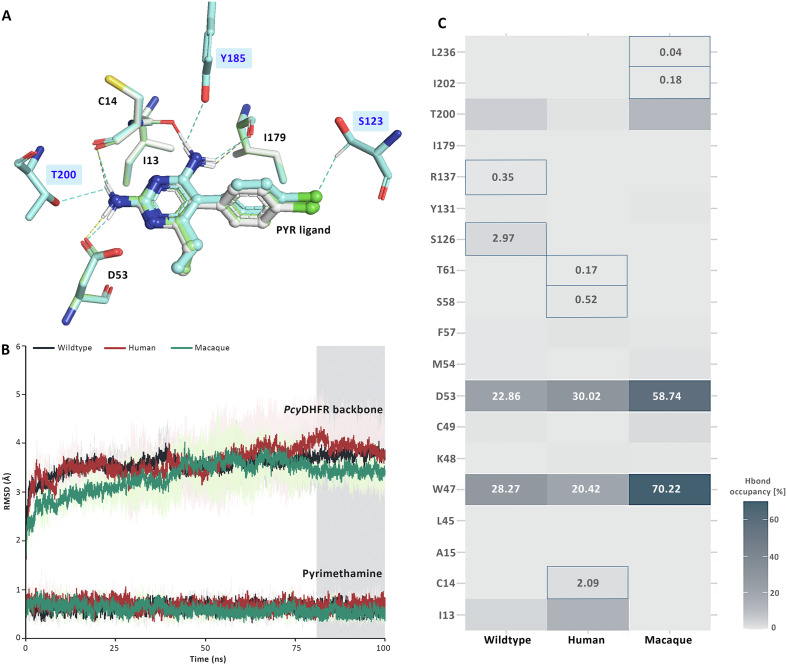
(A) Illustration H-bonding of the docked *Pcy*DHFR-PYR complex of 3 variants *P*. *cynomolgi*. Contact residues are shown in the sticks with grey (wild-type), cyan (human), and light green (macaque). The H bonds are represented in grey, cyan, and yellow dashed lines for wild-type, human, and macaque variants, respectively. A unique hydrogen bond forming residues of the human variant is highlighted in the blue box. (B) Display RMSD analysis of the docked complex through molecular simulations. The last 20 ns were selected for further analysis, indicated in the grey area. (C) Heatmap of the hydrogen occupancy at 81–100 ns of simulation. The amino acids with unique hydrogen occupancy are shown in the square box with their occupancy percentages.

Additional analysis of the highest docking score was executed by protein-ligand interaction analysis. The result revealed that the hydrogen bond interaction between the mutant protein and PYR ligand was slightly different from the wild-type. Moreover, the unique interaction at residues S123, Y185, and T200 was identified in the human *P*. *cynomolgi* (Fig. 3A). The S123 residue was equal to S108 and S117 of *Pf*DHFR and *Pv*DHFR, respectively, directly related to PYR resistance [15, 39]. Consequently, the differences in protein interaction were further confirmed by molecular dynamics (MD) simulation, which determined the percent hydrogen occupancy and calculated binding free energies (Δ*G*_bind_) to elucidate the interaction in detail.

### Molecular dynamic simulation of the *Pcy*DHFR-PYR complex

We simulated the molecular dynamics of the *Pcy*DHFR-PYR complex of the wild-type, human, and macaque variants using GROMACS software, a widely used tool for molecular dynamics simulations. Each complex underwent three 100-ns simulations. The root mean square deviation (RMSD) plots of all variants showed a stable simulation system throughout the 100 ns of simulation (Fig. 3B). The average RMSD scores of the backbone proteins of the wild-type, human, and macaque variants were 3.58, 3.64, and 3.32 Å, respectively. In addition, the stabilizing average of the PYR ligand of these three variants was less than 1 Å with a reasonably low deviation. These values and plots indicate that the *Pcy*DHFR-PYR complex formed a stable structure and system during the simulation. Therefore, the last 20 ns trajectories to analyze hydrogen occupancies and binding free energy.

GROMACS analysis of the three *P*. *cynomolgi* variants mostly detected one hydrogen bond interaction occurring between *Pcy*DHFR and PYR throughout the simulation. The percent hydrogen bond occupancies of 13 wild-type residues ranged from 0.17% to 28.27%. Two of the residues had unique interactions; specifically, S126 and R137 had 2.97% and 0.35% hydrogen occupancy, respectively. Twelve residues of the human variant exhibited hydrogen occupancies ranging from 0.17% to 30.02%; moreover, positions C14, S58, and T61 (found only in this variant), showed 2.09%, 0.52%, and 0.17% hydrogen occupancies, respectively. Remarkably, no occupancies were found at the S123 and Y185 positions, and T200 carried a low occupancy of approximately 1%, consistent with the analysis of the entire simulation of 100 ns. These results suggest that these unique positions from protein interaction analysis might not be involved in the hydrogen bonding of the *Pcy*DHFR-PYR complex in human *P*. *cynomolgi* isolates. In the macaque variant, we observed 0.02%–62.24% hydrogen occupancy across 14 residues of the protein complex. Unique occupancies were observed in I202 (0.18%) and L236 (0.04%). Most notably, the W47 and D53 positions exhibited the highest occupancies among all *P*. *cynomolgi* variants, suggesting that the formation of hydrogen bonds at these residues probably represents a crucial interaction in the *Pcy*DHFR-PYR complex (Fig. 3C).

### Binding free energies of *Pcy*DHFR protein

The simulated complexes from the last 20 ns of the *Pcy*DHFR molecular dynamic were collected and analyzed by MM-PBSA on GROMACS to evaluate the alteration in the binding energy of the mutant variants. Table 4 shows that the main interaction of the *Pcy*DHFR-PYR complex was the Van der Waals interaction, with a highly negative Van der Waals energy (Δ*E*_vdw_), but we observed no significant difference between the *P*. *cynomolgi* variants (*p*-value > 0.05). Similarly, the other binding energies, *i.e.*, electrostatic energy (Δ*E*_ele_), polar solvation energy (Δ*E*_pol_), and solvent accessible surface area (SASA) also showed no significant difference (Table 4). The Δ*G*_bind_ values of these mutants retained similar to wild-type *Pcy*DHFR (*p*-value > 0.05). This result indicates that the detected missense mutations in human and macaque *Pcy*DHFR did not influence PYR-binding affinity; therefore, the PYR drug was predicted to remain effective in treating *P*. *cynomolgi* infection.

**Table 4:** Computational analysis of binding energies of the docked *Pcy*DHFR-PYR complex by using MM-PBSA.

Energies	*Pcy*DHFR binding energies (Kcal/mol ± SD)			*p*-value
	Wild-type	Human	Macaque	
Van der Waals	−32.30 ± 1.14	−31.35 ± 1.37	−32.53 ± 1.03	0.48
Electrostatic	−5.17 ± 1.26	−4.78 ± 2.16	−5.69 ± 1.45	0.81
Polar solvation	23.26 ± 1.71	21.39 ± 0.63	24.03 ± 1.43	0.12
SASA	−3.71 ± 0.03	−3.66 ± 0.06	−3.76 ± 0.07	0.18
**Binding energy**	−**17.92 ± 0.89**	−**18.4 ± 0.31**	−**17.94 ± 0.1**	**0.51**

Note: The significance difference between the three variants of *P*. *cynomolgi* was considered with a *p*-value less than 0.05. Bold text represents the total binding energy of the protein-ligand complex.

### Molecular docking of *Pcy*DHPS with a natural substrate and inhibitors

The structures of *Pcy*DHPS were built and retrieved from SWISS-MODEL, which were established based on a homologous *Pf*DHPS. The ligands were occupied within 3 Å of the protein binding site and reported as ASP fitness score in Table. 3. As a result of the docking score of the substrate, *p*ABA showed a slight difference in mutant variants compared to the wild-type; similarly, the normalized scores scantily exhibited differences as well (approximate 0.01). The protein interaction analysis revealed a unique hydrogen bond interaction between human and macaque *P*. *cynomolgi* at position G551 and K581, which could be a factor in the higher score of the mutant variants.

**Figure 4:**
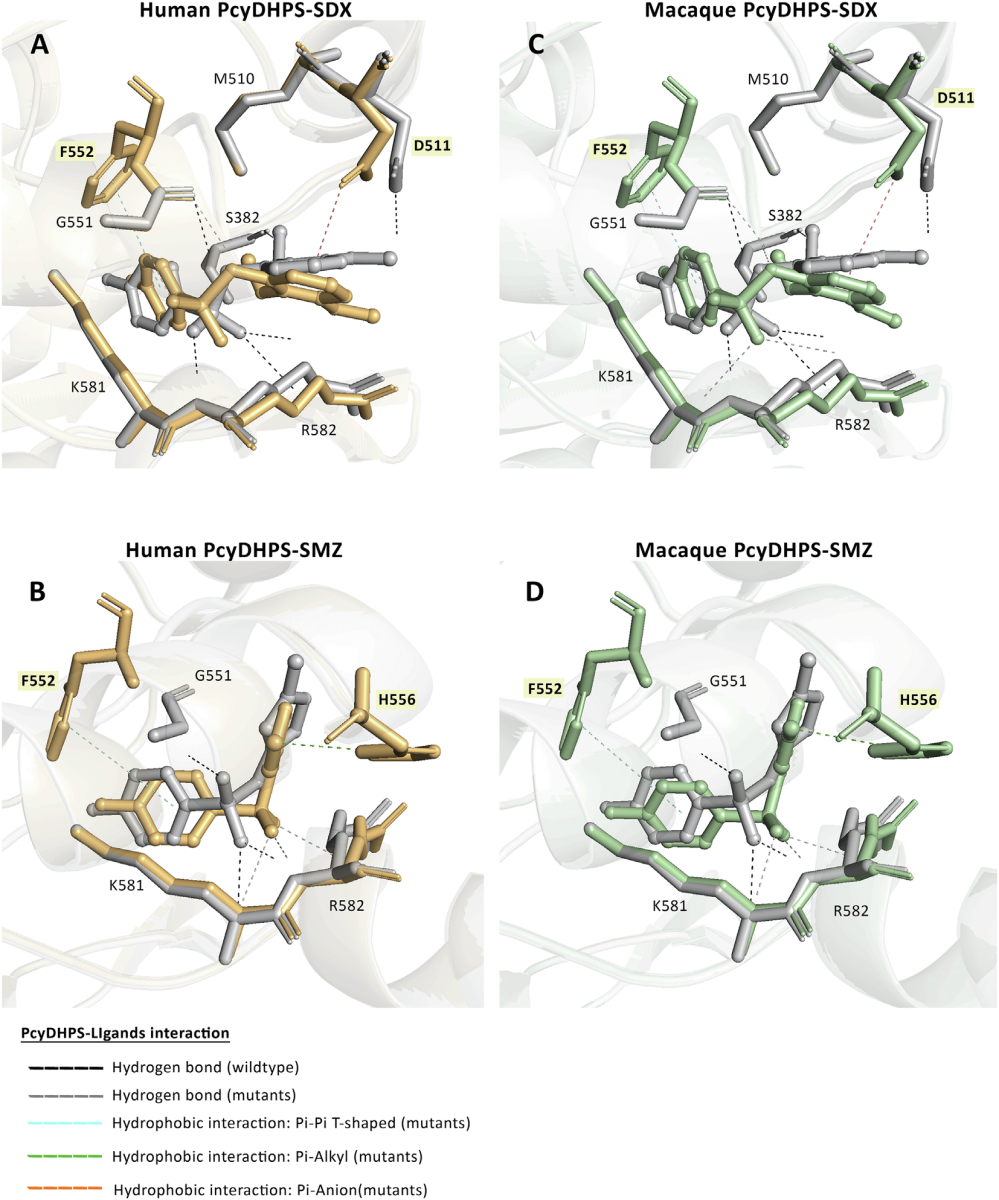
The *Pcy*DHPS-SDX and SMZ complex interaction of human (light orange) and macaque (light green) *P*. *cynomolgi* superimposed with the wild-type interaction (grey). The dashed line represents the interaction between the protein and inhibitors, and the unique interaction residues of mutants are highlighted in yellow boxes.

Meanwhile, the scores of inhibitors SDX and SMZ also exhibited a trivial difference. They barely differed in the normalized score, implying that the mutations found did not alter the antimalarial binding affinity with the *Pcy*DHPS protein. The interaction analysis of the human and macaque *P*. *cynomolgi* protein complexed with SDX showed a hydrophobic interaction with D511 and F552, which was absent in wild-type *P*. *cynomolgi*. Moreover, another hydrogen bond was detected at G551, while only one bond was present in the wild-type (Figs. 4A, 4C). In addition, a unique hydrophobic interaction was found at residue F552 and H556 in the *Pcy*DHPS-SMZ complex of the mutant variants that was absent in the wild-type complex. All *P*. *cynomolgi* variants exhibited three hydrogen bonds with SMZ, a consequence of slightly different docking scores (Figs. 4B, 4D). These results provide insight into how the increase in docking score could be a rationale for the differential bonds in the interaction between *Pcy*DHPS and inhibitors.

Notable, hydrogen bonds interacted with K581 and R582 present in all ligands, implying that these residues were crucial in the interaction between the ligand and *Pcy*DHPS. Nevertheless, complementary experiments are required to investigate the interaction and the impact of mutations on sulfa drug affinity in *P*. *cynomolgi*.

## Discussion

Simian malaria infections have been increasing in Southeast Asia, where long-tailed and pig-tailed macaques (*Macaca nemestrina*) serve as natural hosts to *P*. *knowlesi*, *P*. *cynomolgi*, and *P*. *inui* [4]. This rise is driven by factors such as deforestation, climate change, and the growing geographic overlap between macaque and human populations [14], prompting concern for public health across the region. Although *P*. *cynomolgi* infections in humans and macaques have rarely been reported in Cambodia – with one study noting a 0.96% infection rate in humans (11/14,732) and another reporting 50% prevalence in macaques (27/54) [8, 40] – multiple studies from Thailand have documented such infections more frequently [22, 23, 27]. However, few publications have explored the genotype of antifolate resistance-associated genes, including *pcydhfr* [13] and *pcydhps*, or examined the structural implications of their mutations. To address this gap, we tested all samples using two independent PCR protocols targeting the *18S rRNA* [8] and *cox1* genes [24], both capable of detecting a broad range of simian *Plasmodium* species, including *P*. *knowlesi*, *P*. *coatneyi*, *P*. *inui*, *P*. *fieldi*, and *P*. *cynomolgi*. Notably, both assays consistently detected only *P*. *cynomolgi* mono-infections, with no evidence of co-infections. Sequencing confirmed these findings, aligning with *P*. *cynomolgi* reference sequences. This could reflect regional dominance of *P*. *cynomolgi* at the time of sampling, host- or season-specific factors limiting exposure to other species, or the presence of low-level co-infections below the detection threshold.

Here, we report on four mutations in *pcydhfr* in humans and 24 mutations in macaques infected by *P*. *cynomolgi*. Mutations were detected based on comparisons with the reference sequence of strain M, a high-quality assembly genome that was improved and published in 2017 [20]. Loop-1 of modeled *Pcy*DHFR differed in orientation from *Pf*DHFR, extending from the structure [15, 39]. The S33P mutation was detected in this region. It was equal to one (P33) of six corresponding PYR resistance residues in *P*. *vivax*, which was found in Thai-Myanmar and Thai-Cambodian isolates [32]. However, the location of the S33P mutation was predicted to be far from the catalytic site of *Pcy*DHFR and, consequently, likely has no significant role in drug resistance. Loop-2 presented a different repeat sequence in *Pcy*DHFR from *Pv*DHFR [15]; the relationship between the tandem repeat and protein function remains unclear. The model revealed the location of C49G in human isolates, and N44T and C49S in macaque isolates that were placed close to the binding site of *Pcy*DHFR. The C49 was equal to the C50 of *Pf*DHFR, one of the residues involved in controlling the active site of the protein [39], and related to a large side chain amino acids substitution [11]. Therefore, the mutant models with the mutation at C49 needed to be ascertained initially by computational analysis. The protein docking and interaction analysis of *Pcy*DHFR with six inhibitors revealed no difference between mutants and wild-type, suggesting that the mutations do not affect protein-ligand binding. However, one unique hydrogen bond interacted with the S123 residue of human *Pcy*DHFR-PYR complex that was equivalent to a critical residue conferring PYR and 1CY resistances in *P*. *falciparum* and *P*. *vivax* [15, 34, 39], but the simulation movement displayed no hydrogen occupancy, reflecting the hypothesis that the mechanism of this residue of the parasite was less related to direct hydrogen bond formation to the ligand. During simulation, all *Pcy*DHFR complexes showed a single hydrogen bond formation, and a consistency in the percentage of hydrogen occupancies and contact residues was revealed. The important role of the *Pcy*DHFR’s W47 and D53 residues in hydrogen bonding formation with its ligand was represented by the highest hydrogen occupancy in all *Pcy*DHR-PYR complexes, which correspond to *P*. *falciparum* and *P*. *vivax* [15, 29, 38]. The contact residue of S58 in the human *P*. *cynomolgi* variant was comparable to the positions associated with PYR resistance in *P*. *falciparum* and *P*. *vivax* [9, 35]. Resulting in indirect contact with inhibitors by pointing out to the protein surface and solvent in *Pf*DHFR, whereas *Pv*DHFR is pointed forward into the protein active site [15, 39]. The simulation in this study elucidated the indirect impact of the S58 to PYR binding in *P*. *cynomolgi* by identifying a low percentage of hydrogen bond occupancies and unidentified hydrogen bond occupancies in this position. The VDW interaction was a major stabilizing interaction in the *Pcy*DHFR-PYR complex. It was slightly different and favorable compared to the wild-type of *Pf*DHFR [18]. Interestingly, the total binding energies of all complexes were not significantly different among all variants, confirming that the mutations found in *Pcy*DHFR did not contribute to protein structure and interfere with protein-PYR affinity. However, further experiments should be carried out to confirm this finding.

This study determined that the mutation covered five equivalent residues of the DHPS binding pocket of *P*. *falciparum* and *P*. *vivax*, which confer sulfa drug resistance in the parasite. Among the detected *pcydhps* mutations, the A585V mutation of macaque *P*. *cynomolgi* was equal to the fifth significance residues, A613 and V585, respectively [10, 35]. In an *in vitro* study of *Pv*DHPS, the V585A mutation resulted in a 2-fold increase in the *K*_i_ value of SDX, while the A383G mutation led to a 30-fold increase that is consistent with *Pf*DHPS. This implies that the mutation at the fifth position of parasites has a low influence on the SDX binding affinity compared to the mutated residues at the first and second positions. The fifth position indirectly affects protein interaction by contributing to salt-bridge formation, which helps dimerization of the protein [2, 21, 36]. Although *Pcy*DHPS is closely related to *Pv*DHPS and has 90% protein sequence similarity with the available structure, the structure contains a constriction and an active site that limits the accessibility of SDX and SMZ ligands for docking in this study; accordingly, *Pf*DHPS was selected as the template structure. Loop-1 of the build model *Pcy*DHPS was located far from the core folding structure, and another loop contained a tandem-like repeat, like in *Pv*DHPS, which has a function in *Pv*HPPK-DHPS protein dimerization, providing evidence of indirect function on protein activity [36]. Molecular docking of the *Pcy*DHPS mutant variants is only slightly different from that of the wild-type. The higher docking score of the mutant variants is likely due to a unique hydrophobic interaction involving five residues to support ligand binding and stabilize ligand alignment along the binding site of *P*. *falciparum*. All *Pcy*DHPS variants formed a hydrogen bond with K581 and R582, which could contribute, as characterized in *Pf*DHPS, to help the ligand to stay in the proper position and form a salt bridge to secure the substrates at the binding site [2]. The interaction analysis of *Pcy*DHPS variants revealed the persistence of the likely crucial residue for interaction between protein and inhibitors, proposing that the detected mutations in *P*. *cynomolgi* do not alter *Pcy*DHPS-ligand binding. This suggests the potential of sulfa drugs for treating *P*. *cynomolgi*, but further testing is required to confirm the relationship between these mutations and sulfa drug resistance in parasites.

The mutations in *pcydhfr* and *pcydhps* should be investigated further to clarify the relationship between these polymorphisms and antifolate resistance in *P*. *cynomolgi*. The haplotype pattern of the *pcydhfr*-*pcydhps* was distinct among different hosts, indicating that transmission of *P*. *cynomolgi* among hosts could be limited, or the parasite has been circulating within the host population and area. To investigate this hypothesis, an extensive sample size analysis is required to confirm a significant comparison of the results between the hosts.

The relatively high number of amino acid differences observed between the human and simian *P*. *cynomolgi* isolates may reflect natural genetic divergence rather than selective pressure from antimalarial drugs. Several of these polymorphisms were found in regions not previously associated with resistance phenotypes and may instead represent neutral mutations or lineage-specific variations. One possible explanation, particularly regarding the *DHFR* gene, is that a single zoonotic crossover event may have occurred in the past, followed by local expansion and sustained circulation within the human population. The human-infecting isolates may represent a genetically distinct subpopulation that has at least partially adapted to human hosts, leading to reduced gene flow with macaque parasite populations. Alternatively, these differences could indicate a cryptic transmission cycle or a founder effect, where a limited number of genetically distinct parasites crossed into humans and expanded clonally. These hypotheses need further investigation using whole-genome sequencing and broader geographic or longitudinal sampling.

In conclusion, the observed amino acid differences between human and simian *P*. *cynomolgi* isolates are more likely to reflect natural genetic divergence than antimalarial drug pressure. These variations may result from a past zoonotic crossover event, followed by local adaptation and limited gene flow with macaque populations, or may reflect a cryptic transmission cycle. Further investigation using whole-genome sequencing and expanded sampling is needed to clarify the evolutionary and epidemiological significance of these findings.

## Data Availability

All data generated or analyzed during this study are included in this published article.

## Supplementary material

*Supplementary File 1*: Nucleotide sequences of pcydhfr. *Supplementary File 2*: Nucleotide sequences of pcydhps. The supplementary material for this article can be found at https://www.parasite-journal.org/10.1051/parasite/2025057/olm.
